# Changes in the contents of selected polycyclic aromatic hydrocarbons in soils of various types

**DOI:** 10.1007/s11356-014-3901-9

**Published:** 2015-01-15

**Authors:** Magdalena Banach-Szott, Bozena Debska, Alicja Wisniewska, Jaroslaw Pakula

**Affiliations:** Department of Environmental Chemistry, University of Technology and Life Sciences, Bernardynska St. 6, 85-029 Bydgoszcz, Poland

**Keywords:** Polycyclic aromatic hydrocarbons, Soils, Soil organic matter, Decomposition, HPLC

## Abstract

The aim of the paper was to determine the stability and the decomposition intensity of selected polycyclic aromatic hydrocarbons (fluorene, anthracene, pyrene, and chrysene) in soils that are under agricultural use. Soil was sampled from the arable layer that is representative of the Kujawy and Pomorze Provinces, which are located in the northwestern part of Poland. The soil samples were polluted with selected PAHs at an amount corresponding to 10 mg PAHs/kg. PAH-polluted soil samples were incubated for 10, 30, 60, 120, 180, and 360 days at a temperature of 20–25 °C and a fixed moisture of 50 % field water capacity. High-performance liquid chromatography (HPLC) was used to determine the content of PAHs. It was found that the process of the degradation of PAHs was most intensive during the first 30 days of the experiment; however, three-ring PAHs (fluorene and anthracene) definitely decomposed faster than the four-ring ones (pyrene and chrysene). The results also confirm the significant role of soil organic matter in sorption and activation processes of PAHs.

## Introduction

Polycyclic aromatic hydrocarbons (PAHs) are compounds that occur in the air (Lee et al. 1981), water (Renner et al. 1997), and, mostly, in soil and sediments (De Voogt et al. 1996; Martens et al. 1997; Schantz et al. 1997). Soils polluted with PAHs pose a serious problem, and for that reason, the Communication from the Commission to the Council and the European Parliament, the European Economic and Social Committee and the Committee of the Regions: toward a thematic strategy for soil protection (COM (2002), 179 final) defines eight major threats to soils in the European Union, including a decrease in the content of organic matter and soil contamination. Special attention was paid to the role of organic matter in maintaining the key soil functions and the content of carbon as a key component of organic matter. It was also found that the occurrence of organic compounds in soil, including PAHs, at a concentration exceeding some levels, can result in soil degradation.

PAHs that occur in the soil environment can be of an anthropogenic or natural origin (Johnsen and Karlson 2007; Jones et al. 1989; Maliszewska-Kordybach 1999). However, as for total contamination, the amounts of PAHs derived from natural sources are negligible as compared with the amount resulting from human activity (Maliszewska-Kordybach et al. 2008). The content of PAHs in soils falls within a wide range, depending on the place, type, and the method of soil use. The highest level of PAHs is found in the soils of large cities, along roads and in the vicinity of industrial plants (Adamczewska et al. 2000; Jones et al. 1989; Wild and Jones 1995) whereas the content of PAHs in arable and meadow soils as well as in the soils of other agricultural land usually does not exceed a few hundred microgram per kilogram (Maliszewska-Kordybach et al. 2008; Menzie et al. 1992; Wild and Jones 1995; Maliszewska-Kordybach et al. 2010).

The decomposition rate of PAHs in soils depends on environmental factors (moisture, pH, temperature, oxygen access) (Bossert and Bartha 1986; Chiou 1989; In Der Wiesche et al. 2003; Lors et al. 2012; Maliszewska-Kordybach 1993; Schlegel 2005), microbiological factors (the occurrence of populations of fungi, bacteria, and Actinobacteria), and on the physicochemical properties of respective compounds (Mackay et al. 1992; Sutherland et al. 1995; Weiss et al. 1994).

PAHs are considered to be “permanent organic pollutants” with a high capacity for bioaccumulation and a low susceptibility to degradation. They also demonstrate toxic, mutagenic, and carcinogenic properties (Maliszewska-Kordybach 1999; Zhang et al. 2005). Those compounds undergo many abiotic transformations in soil (sorption, volatilization, photodegradation, leaching, chemical decomposition, etc.) as well as biotic ones (microbiological decomposition) (Chiou 1989; Lors et al. 2012; Menzie et al. 1992; Revitt et al. 2014).

As has been reported in the literature (Atanasova and Brümmer 2004; Maliszewska-Kordybach 1993; Ni et al. 2008; Yang et al. 2010; Pan et al. 2006; Plaza et al. 2009; Wang et al. 2011), the decomposition, and therefore the content of PAHs in soils, depends on the content and the quality of organic matter to a significant degree, thus showing a high sorption potential (Chen et al. 2007; Fries 1995; Kastner et al. 1999; Kohl and Rice 1998; Nieman et al. 1999; Pan et al. 2006; Pignatello and Xing 1996; Richnow et al. 1997; Yang et al. 2010). It is generally assumed that humins show a much greater sorption potential toward PAHs than humic acids. Humic acids demonstrated a greater PAH sorption capacity than fulvic acids (Pan et al. 2006). The effect of the high sorption capacity by organic matter toward PAHs is limited by both the migration of those compounds deep in the soil profile (Conte et al. 2001; Oleszczuk and Baran 2005; Petruzzelli et al. 2002; Yang et al. 2010) and their availability to microorganisms (Bauer and Capone 1985; Cousins et al. 1997; Jensen and Folker-Hansen 1995).

Among the key factors that determine the stability of PAHs in soils are their structure and properties. The two- and three-ring hydrocarbons, which have a lower molecular weight and a higher solubility in water, are more susceptible to degradation and photodegradation than the four-ring hydrocarbons. An increase in the number of rings in the molecules of PAHs increases their molecular weight and hydrophobic properties, thus limiting microbiological decomposition (Fries 1995; Kohl and Rice 1998).

Due to the ongoing and more rapid economic development as well as increasing environmental pollution with PAHs, it is important to determine the decomposition rate of those compounds as well as the factors that affect their stability.

The aim of the present paper was to determine the stability and the intensity of the rate of the decomposition of selected PAH (fluorene, anthracene, pyrene, and chrysene) in various types of soils.

## Materials and methods

### Reagents

Standard mixtures of PAHs (US EPA 16 components), in acetonitrile, high-performance liquid chromatography (HPLC) purity, were provided by Dr. Ehrenstofer GmbH.

Fluorene, anthracene, pyrene, and chrysene, in acetonitrile, HPLC purity, provided by LGC Standards.

Fluorene, chrysene, and pyrene, analytical standard, provided by Cerilliant.

Anthracene, analytical standard, provided by Riedel-de Haën.

Acetonitrile, cyclohexane, and dichloromethane, HPLC purity, provided by Avantor Performance Materials, Poland.

Water was purified using the Millipore Milli-Q system.

### Soil samples

Soil was sampled from the arable layer that is representative of the Kujawy and Pomorze Regions (Poland). The following soils were investigated: *Gleyic Phaeozem* that was sampled in the vicinity of Inowrocław (Ph1) and at Orlinek (Ph2), *Haplic Arenosol* (Hp1) that was sampled in Bydgoszcz and *Mollic Fluvisols* with the texture of sandy loam (FL1), and *Gleyic Fluvisol* with the texture of fine sand (FL2) that was sampled in the vicinity of Bydgoszcz. The basic properties of the soil samples and the fraction composition of humus are given in Tables 1 and 2 (Debska et al. 2011, 2012). The content of selected PAHs in soils before their pollution is given in Table 3.

**Table 1:** Basic parameters of soils

Soil	pH in H_2_O	TOC (g kg^−1^)	Nt (g kg^−1^)	TOC/Nt	Grain size composition (%)		
					2–0.05 mm (FR1)	0.05–0.002 mm (FR2)	<0.002 mm FR3)
Gleyic Phaeozems (Ph1W)	5.9	25.37	1.91	13.28	67	20	13
Gleyic Phaeozems (Ph2W)	6.3	21.23	1.75	12.13	73	16	11
Haplic Arenosols (Hp1W)	6.4	5.72	0.42	13.62	93	4	3
Mollic Fluvisols (FL1W)	6.4	34.02	2.69	12.65	55	29	16
Gleyic Fluvisols (FL2W)	6.3	8.17	0.63	12.97	82	12	6

From Debska et al. (2011, 2012). Grain size composition was determined applying the areometric method

*TOC*, total organic carbon, *Nt* total nitrogen, determined with the analyzer vario MAX CNS (Elementar, Germany)

**Table 2:** Fractional composition of soils humus

Soil	DOC	C_deka_	C_HAs_	C_FAs_	C_HUMIN_ (%)	C_HAs_/C_FAs_
	(mg kg^−1^)					
Gleyic Phaeozems (Ph1W)	152.6	1061	7523	3977	50.49	1.89
Gleyic Phaeozems (Ph2W)	120.5	1675	6997	4673	37.14	1.50
Haplic Arenosols (HpW)	68.3	350.0	1753	1537	36.36	1.14
Mollic Fluvisols (FL1W)	191.0	2145	7559	3701	60.60	2.04
Gleyic Fluvisols (FL2W)	87.1	1360	1895	2005	35.62	0.94

From Debska et al. (2012)

*DOC* dissolved organic carbon extracted with 0.004 M CaCl_2_ (Debska et al. 2012), *C*_*deka*_ carbon in solutions after decalcification, *C*_*HAs*_ carbon of the fraction of humic acids, *C*_*FAs*_ carbon of the fraction of fulvic acids, *C*_*HUMIN*_ carbon of the humin fraction, determined according to the Schnitzer methods (Debska et al. 2012)

**Table 3:** Contents of PAHs in the soil samples (prior to their additional pollution)

Sample	Fluorene	Anthracene	Pyrene	Chrysene	Sum
	(μg kg^−1^)				
Ph1W	115	10.0	43.2	146	314
Ph2W	56.2	0.774	16.9	12.6	86.5
Hp1W	3.87	0.128	3.75	0.855	8.60
FL1W	115	6.59	106	42.3	270
FL2W	91.5	0.814	17.0	7.80	117

The soil samples (1 kg) were placed into glass containers, and they were polluted with selected PAHs (fluorene, anthracene, pyrene, and chrysene). The containers were covered with a porous cap which enabled oxygen pass through. Soil samples with moisture 50 % of field water capacity (FWC) were poured into the mixtures of those PAHs at a total amount corresponding to 10 mg PAHs/kg of soils (2.5 mg of each of the selected PAHs). The solid PAHs were dissolved in dichloromethane. PAH-polluted soil samples were incubated for 10, 30, 60, 120, 180, and 360 days at a temperature of 20–25 °C and a fixed moisture (50 %). Water content was gravimetrically corrected every 3 days. After a specific incubation time, the entire samples were liquidated. The incubation was performed in three reps for each sampling date. After the incubation, the soil samples were dried at room temperature, mixed, and screened through a 2-mm-mesh sieve.

### PAH extraction from soils

The extraction of PAHs was done for the initial samples and after 10, 30, 60, 120, 180, and 360 days of incubation. Soil samples were extracted with cyclohexane at a ratio of 1:25 (*w*/*v*) for 3 h using a Soxhlet apparatus that was provided by Behr Labor-Technik. The extracts were evaporated down to dryness, and the residue was dissolved in acetonitrile (ACN).

### Determining the content of PAHs in the extracts

The content of PAHs in the extracts was assayed using the HPLC Series 200 equipped with the DAD (absorption) that was provided by PerkinElmer and an FL (fluorescence) detector. The analytical column to separate PAHs provided by Waters was used (250 × 4.6 mm, 5 μm). The mobile phase was composed of eluent A: H_2_O and eluent B: ACN. A gradient separation program of a varied flow rate was used. The initial composition of the mobile phase was 70 % of eluent B, and its concentration was increased linearly throughout the analysis. The gradient was completed after 34 min when the content of eluent B reached 100 %.

The extracts of the unpolluted (initial) soil samples were analyzed using a fluorescence detector at an excitation wavelength of *λ*_ex_ = 250 nm and emissions of *λ*_em_ = 405 nm. The injection was 10 μL.

The extracts of the samples of soils that had been polluted with PAHs and incubated for 10, 30, 60, 120, 180, and 360 days were analyzed using an absorption detector. The detection was done at *λ* = 254 nm. The injection was 100 μL.

The qualitative analysis of respective hydrocarbons was performed by comparing the soil extract chromatograms with the chromatograms of the model mixtures.

The quantitative assays of fluorene, anthracene, pyrene, and chrysene were done based on their model curves.

### Defining the half-life—*T*_1/2_

In order define the half-life, the reaction rate constant for pseudo-first order there was calculated using the following equation:

$$ \ln \frac{C_o}{C_t}=k\cdot t $$

where

$$ \begin{array}{c}\hfill k\hbox{--} \mathrm{apparent}\ \mathrm{reaction}\ \mathrm{rate}\ \mathrm{constant}\ \mathrm{of}\ \mathrm{the}\ \mathrm{pseudo}-\mathrm{first}\ \mathrm{order}\ \left(1/\mathrm{day}\right),\hfill \\ {}\hfill t\hbox{--} \mathrm{time}\left(\mathrm{days}\right),\hfill \\ {}\hfill {c}_0\hbox{--} \mathrm{initial}\ \mathrm{content}\ \mathrm{of}\ \mathrm{PAHs}\ \mathrm{in}\ \mathrm{the}\ \mathrm{soil},\hfill \\ {}\hfill {c}_{\mathrm{t}}\hbox{--} \mathrm{content}\ \mathrm{of}\ \mathrm{PAHs}\ \mathrm{in}\ \mathrm{the}\ \mathrm{soil}\ \mathrm{after}\ \mathrm{time}\ t,\hfill \end{array} $$

Then, the half-life (*T*_1/2_) was calculated using the following equation:

$$ {T}_{1/2}=\frac{ \ln 2}{k} $$

### Statistical analyses

The similarities across the objects were evaluated using a cluster analysis. The relationships between the features were defined using the coefficients of correlation. The above relationships were determined using STATISTICA MS statistics software.

## Results and discussion

### Characterization of soil samples

Very important in the process of the sorption of PAHs by organic matter is its general content as well as quality composition. The main fraction of organic matter that determines the detoxification properties in regard to PAHs are the humic substances (Atanasova and Brümmer 2004; Chen et al. 2007; Chiou 1989; Ni et al. 2008; Wang et al. 2011; Yang et al. 2010). The processes of the sorption of PAHs by organic matter also depend on the properties of PAHs themselves. Some authors (Fries 1995; Kohl and Rice 1998), when comparing the sorption of fluorene, which represents three-ring PAHs and five- and six-ring compounds, found a greater share in the sorption of humic and fulvic acids than humins for fluorene, which is due to the small size of the fluorene molecule and its greater solubility in water, which accounts for its capacity to form bonds with humic and fulvic acids, which are not accessible to PAHs with a greater number of rings. Pyrene is bonded by humins and humus acids to the same extent (Nieman et al. 1999). Anthracene, on the other hand, is sorbed almost evenly by all of the fractions of organic matter (Kastner et al. 1999; Pignatello and Xing 1996). Based on this information, one can state that compounds with a high molecular weight and a lower solubility in water undergo sorption by the fraction of humins to a greater degree, while hydrocarbons that have a higher solubility are bonded more strongly by humic and fulvic acids (Kastner et al. 1999; Meyer and Steinhart 2011). Additionally, it was shown that humic acids limit the movement of hydrocarbons deep in the soil profile, while fulvic acids stimulate that process (Petruzzelli et al. 2002).

Based on the data presented in Tables 1, 2, 3, and 5, it was found that the highest content of PAHs in the initial soil samples (before pollution) that were analyzed was observed in the soils with the highest content of total organic carbon (TOC), total nitrogen (Nt), dissolved organic carbon (DOC), and *C*_HAs_ fractions as well as the highest share of the clay fraction and *C*_HUMIN_ fraction and the highest values of the ratio of *C*_HAs_/*C*_FAs_ (Ph1W and FL1W). The lowest content of PAHs investigated; the content of TOC, Nt, DOC, fraction *C*_deka_, *C*_HAs_, and *C*_FAs_; and the share of clay fraction, on the other hand, were identified in Haplic Arenosols (Hp1W) (Tables 1 and 2). Similar relationships were reported by Menzie et al. (1992) and Wild and Jones (1995).

### Changes in the content of selected PAHs in soils during incubation

Table 4 presents the changes in the content of fluorene, anthracene, pyrene, and chrysene observed for the soil samples during the incubation. It was found that the content of PAHs that were analyzed in the soil samples decreased throughout the experiment. However, the highest decomposition of those compounds occurred during the initial time of incubation (0–10 and 11–30 days). The highest decomposition rate over 10 days, irrespective of the soil type, was recorded for anthracene. In the Gleyic Phaeozems with a higher content of TOC (Ph1), the anthracene content decreased by 88.2 and by 86.7 % of the initial content in the Haplic Arenosol sample (Table 4). The content of anthracene in the other soil samples after 10 days of incubation accounted for, on average, 27.9 % of the initial content. The lowest rate of decomposition among PAHs that were investigated was noted for chrysene for which the decrease in the content after 10 days ranged from 16.3 % (sample number FL2-10) to 44.9 % (sample number Hp1-10). As compared to chrysene, pyrene decomposed at a faster rate, except for the sample Mollic Fluvisols with the texture of sandy loam (FL1) in which the content of PAHs accounted for 85.8 % of the initial content after 10 days of incubation. The other soil samples showed a similar decrease in the content of pyrene—on average by 49.2 % as compared with the initial content. As for fluorene, the fastest decomposition was reported for sample Haplic Arenosols, which had an 80.3 % decrease in content. The smallest decrease in the content of fluorene was recorded for Gleyic Phaeozems (Ph1 and Ph2)—on average by 29.0 % of the initial content. A faster decomposition of fluorene was reported in *Mollic* and Gleyic Fluvisols (FL1 and FL2) in which the content of that PAH accounted for 37.3 and 41.1 % of the initial content after 10 days of incubation, respectively (Table 4).

**Table 4:** Changes in the content of the selected PAHs during incubation

Sample	Fluorene	Anthracene	Pyrene	Chrysene
	(% of the initial content)			
Gleyic Phaeozems				
Ph1W’^a^	100	100	100	100
Ph1-10^b^	73.0	11.8	44.0	68.8
Ph1-30	35.0	5.4	33.0	36.5
Ph1-60	12.3	4.4	20.6	20.0
Ph1-120	9.2	3.6	17.5	17.8
Ph1-180	7.0	2.6	11.2	11.9
Ph1-360	5.2	1.9	7.1	8.0
Gleyic Phaeozems				
Ph2W’	100	100	100	100
Ph2-10	69.0	28.0	53.2	70.2
Ph2-30	14.5	7.2	18.7	23.0
Ph2-60	7.8	6.9	13.6	13.2
Ph2-120	6.9	4.6	8.8	8.5
Ph2-180	4.0	2.9	6.8	7.3
Ph2-360	3.4	0.74	5.0	6.0
Haplic Arenosols				
Hp1W’	100	100	100	100
Hp1-10	19.7	13.3	54.3	55.1
Hp1-30	10.5	6.0	39.7	25.0
Hp1-60	8.9	4.8	29.2	26.7
Hp1-120	7.3	3.3	21.5	18.9
Hp1-180	3.8	2.4	11.2	17.6
Hp1-360	2.2	0.71	4.6	16.5
Mollic Fluvisols				
FL1W’	100	100	100	100
FL1-10	62.7	30.3	85.8	77.4
FL1-30	26.0	5.7	37.5	28.2
FL1-60	7.8	5.1	19.5	16.5
FL1-120	6.0	2.8	12.0	15.5
FL1-180	4.2	2.1	9.3	14.2
FL1-360	3.4	1.4	6.7	11.2
Gleyic Fluvisols				
FL2W’	100	100	100	100
FL2-10	58.9	25.3	51.8	83.7
FL2-30	15.3	9.5	26.9	29.9
FL2-60	11.6	8.7	24.8	29.1
FL2-120	6.9	3.2	18.7	28.1
FL2-180	5.0	3.0	12.4	14.8
FL2-360	3.6	1.3	3.6	9.3

^a^1W’ initial soil, directly after pollution

^b^Period of incubation (days)

Drawing on the results, it was also found that the sample of Gleyic Phaeozems (Ph1) showed the largest decrease in the content of anthracene and pyrene (Table 4). Fluorene and chrysene, on the other hand, were decomposed more quickly in the Haplic Arenosol sample and demonstrated the lowest content of TOC and Nt as well as the lowest share of clay fraction. In the sample marked with the symbol Hp1-10, a decrease in fluorene and chrysene by 80.3 and 44.9 % of the initial content was noted, respectively (Table 4). The lowest rate of decomposition of anthracene and pyrene in the first 10 days of incubation was observed for the sample of Mollic Fluvisols with the texture of sandy loam (sample number FL1); their content accounted for 30.3 and 85.8 % of the initial content, respectively (Table 4). Interestingly, the sample of Mollic Fluvisols with the texture of sandy loam demonstrated the highest content of TOC and Nt as well as the highest share of clay fraction. Fluorene decomposed at the lowest rate in Gleyic Phaeozems (Ph1) and chrysene in the sample of Gleyic Fluvisols with the texture of fine sand (Table 4).

In the next period of the experiment (11–30 days), a further decrease was observed in the content of PAHs analyzed (Table 4). The most intensive decomposition of anthracene and pyrene was observed in the sample of Mollic Fluvisols with the texture of sandy loam (FL1). The content of anthracene from the 11th to the 30th day of incubation decreased by 24.6 % and pyrene by 48.3 %. Chrysene was decomposed at the fastest rate in the sample of Gleyic Fluvisols with the texture of fine sand—a decrease in the content by 53.8 %; however, interestingly, a very similar decomposition rate of that PAH was recorded for *Mollic Fluvisols* with the texture of sandy loam (FL1) and Gleyic Phaeozems with a lower content of TOC (Ph2) (Tables 1 and 4). Gleyic Phaeozems (sample number Ph2) also revealed the greatest decrease in the content of fluorene—a 54.5 % decrease. The lowest decomposition rate for fluorene and chrysene, from the 11th to the 30th day of incubation, was recorded in the samples of Haplic Arenosols (Table 4). The Haplic Arenosol samples also showed a slow decomposition of anthracene and pyrene; however, the slowest decomposition rate of those PAHs occurred in the sample of Gleyic Phaeozems with a higher content of TOC (Table 4, Ph1).

### Changes in half-life of PAHs in soils in the period of 0–30 days

**Fig. 1:**
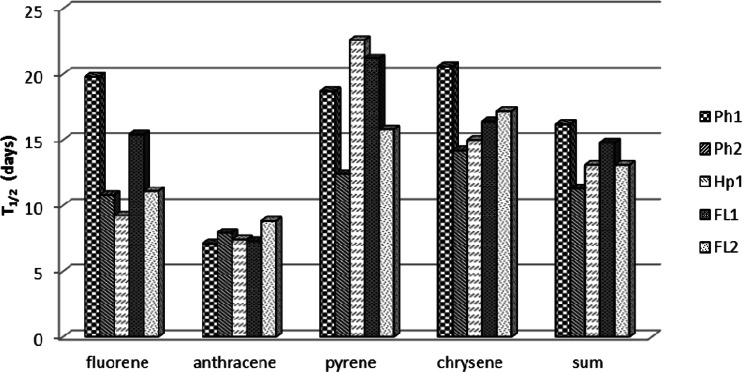
Half-life (*T*_1/2_) of the PAHs in soils in the period of 0–30 days

Based on the calculations of the data from the first 30 days, the half-life confirmed that the highest rate of decomposition was reported for anthracene. The half-life for anthracene fell within a range of 7.11 to 8.82 days. The slowest decomposition rate was reported for chrysene and pyrene; the half-life for those PAHs was, on average, 16.68 and 18.14 days, respectively (Fig. 1).

**Fig. 2:**
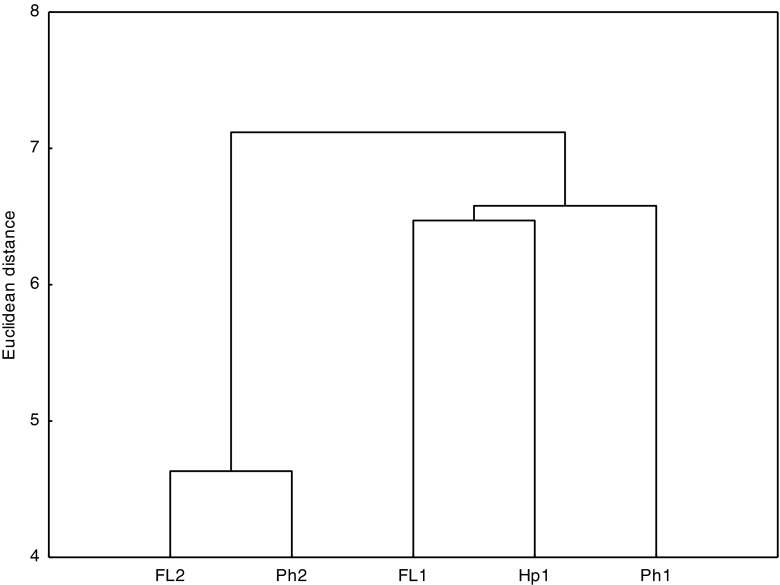
Cluster analysis of soils determined based on half-life (*T*_1/2_) of the PAHs calculated for the period of 0–30 days

From the results that were recorded, one can assume that the decomposition rate of PAHs throughout the first 30 days of incubation depended on the properties of PAHs more than on the properties of the soils themselves, which is confirmed by the lack of correlation between the half-life and the basic properties (parameters) of the soil as well as the results of the cluster analysis (Table 5, Fig. 2). The objects with similar properties are located in homogenous groups on the dendrograms. The dendrogram, produced on the basis of the half-life over the period 0–30 days, differed between two major groups. The first one was made up of the samples of Gleyic Phaeozems (Ph2) and Gleyic Fluvisols with the texture of fine sand (FL2), while the second one was composed of samples of *Mollic Fluvisols* with the texture of sandy loam, Haplic Arenosol, and Gleyic Phaeozems (Ph1). For that reason, a similar decomposition rate was recorded for the soil samples that differed in their physicochemical properties.

**Table 5:** Significant correlations between soil quality parameters and the content of the total PAHs in the initial samples and half-life (*T*_1/2_)

Parameter	Sum of PAHs^a^	*T*_1/2_ (0–30 days)	*T*_1/2_ (30–180 days)	*T*_1/2_ (0–360 days)
TOC (g kg^−1^)	0.8101	–	−0.9453	–
Nt (g kg^−1^)	0.7760	–	−0.9406	–
*C*_deka_ (mg kg^−1^)	–	–	–	–
*C*_HAs_ (mg kg^−1^)	0.7355	–	−0.9962	–
*C*_FAs_ (mg kg^−1^)	0.5356	–	−0.9082	–
*C*_HUMIN_ (%)	0.8495	0.7303	−0.7490	–
FR1 (%)	−0.8503	–	0.8863	–
FR2 (%)	0.8461	–	−0.8447	–
FR3 (%)	0.8430	–	−0.9444	–
*C*_HAs_/*C*_FAs_	0.8187	0.6032	−0.9436	–

*TOC* total organic carbon; *Nt* total nitrogen; *C*_*deka*_ carbon in solutions after decalcification; *C*_*HAs*_ carbon of the fraction of humic acids; *C*_*FAs*_ carbon of the fraction of fulvic acids; *C*_*HUMIN*_ carbon of the humin fraction; *FR1*, *FR2*, *FR3* percentage share of respective fractions: 2–0.05 mm, 0.05–0.002 mm, and <0.002 mm

^a^Sum of fluorene, anthracene, pyrene, and chrysene in the soil samples prior to their additional pollution (data in Table 3)

According to In Der Wiesche et al. (2003), Lors et al. (2012), and Maliszewska-Kordybach (1993), microbiological processes are the main reason for the significant decreases in the content of PAHs during the initial incubation period. The degradation of microbiological PAHs in soil, on the other hand, is conditioned by, e.g., moisture and temperature. As reported by Bossert and Bartha (1986), the optimal soil moisture for the fungi and bacteria that decompose PAHs is from 37 to 65 % FWC, while the temperature that is optimal for the development of soil microflora is 15–38 °C (Lors et al. 2012; Maliszewska-Kordybach 1993; Schlegel 2005). The experimental conditions thus correspond to the conditions that are optimal for the development of the fungi and bacteria that decompose PAHs .

One of the factors that determined the decomposition rate over the initial experiment period was the properties and the structure of PAHs that were analyzed. Fluorene and anthracene revealed a higher rate of decomposition, as compared to pyrene and chrysene; after 30 days of incubation, there was a decrease in the content of anthracene in all of the soils by an average of 93.2 % of the initial content and fluorene by 79.7 %, chrysene by 71.5 %, and pyrene by 68.8 % of the initial content (Table 4). Kohl and Rice (1998) and Lors et al. (2012) showed that the microbiological decomposition of hydrocarbons occurs faster for compounds with a lower molecular weight and greater solubility .

The greater resistance to decomposition in pyrene and chrysene, as compared to fluorene and anthracene, may be also due to the pattern of rings, which show a high thermodynamic stability (Mackay et al. 1992). Also interestingly, four-ring hydrocarbons, including pyrene and chrysene, are sorbed by organic matter more strongly, which, as a result, limits the bioavailability and leaching of those PAHs (Bauer and Capone 1985; Conte et al. 2001; Cousins et al. 1997; Revitt et al. 2014). The high stability of pyrene and chrysene in the soil samples analyzed is also connected with their low susceptibility to oxidation, which is seen from the low vapor pressure and low solubility in water (Mackay et al. 1992).

On the other hand, a large decrease in the content of fluorene and anthracene in the soil samples investigated over the initial incubation period, unlike pyrene and chrysene, could have been connected with their oxidation, which is seen from the values of Henry’s constants (H), which range from 10^−5^ to 10^−3^ atm mol^−1^ m^−3^, which is classified as the range for compounds of moderate volatility (de Gert-Jan et al. 1998; Mackay et al. 1992).

### Changes in half-life of PAHs in soils in the period of 30–180 days

**Fig. 3:**
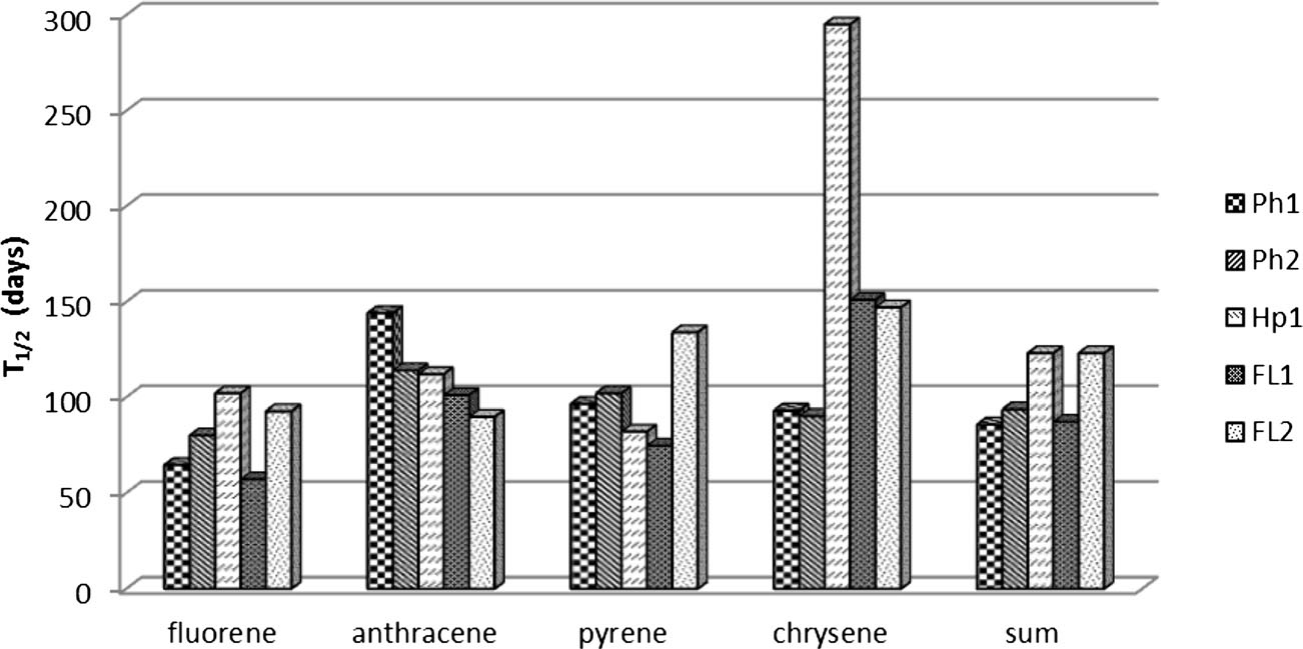
Half-life (*T*_1/2_) of the PAHs in soils in the period of 30–180 days

The content of PAHs analyzed decreased in the successive stages of the experiment. The lowest content after 180 days of incubation was found for anthracene; its content ranged from 2.1 to 3.0 % of the initial content (Table 4). The time of half-life of anthracene embraced the period from 101 to 144 days. On average, the lowest *T*_1/2_ was recorded for fluorene (79.3 days) and the highest for chrysene decomposition in Haplic Arenosols, *Mollic Fuvisols*, and Gleyic Fluvisols. The fluorene content in the soil samples after 180 days was, on average, 4.8 % of the initial content and pyrene 10.2 % and chrysene 13.1 % of the initial content (Fig. 3).

**Fig. 4:**
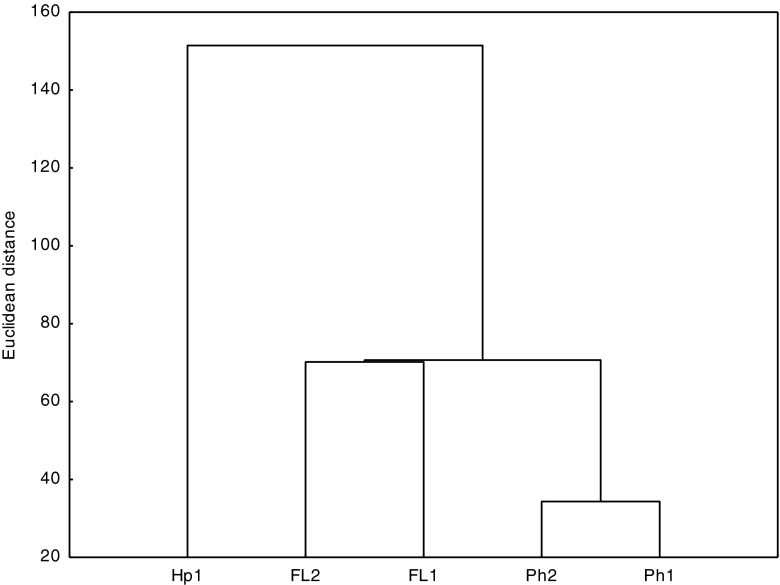
Cluster analysis of soils determined based on half-life (*T*_1/2_) of the PAHs calculated for the period of 30–180 days

The decomposition rate of PAHs over 30–180 days depended on the physicochemical properties of the soils, which is seen from the correlations between the half-life and the soil quality parameters (Table 5) and correlations showed in dendrogram (Fig. 4). The highest *T*_1/2_ values that were recorded for the sum of PAHs that were investigated were observed for Haplic Arenosols and Gleyic Fluvisols with the texture of fine sand and thus showed the lowest content of TOC, Nt, DOC, *C*_deka_, *C*_HAs_, and *C*_FAs_ and the lowest value of the ratio *C*_HAs_/*C*_FAs_ as well as the lowest share of clay fraction of all those researched. In the dendrogram obtained on the basis of the half-life, two groups could be distinguished to belong to one of them *Mollic* and Gleyic Fluvisols, and the second one consists of Gleyic Phaeozems.

### Changes in half-life of PAHs in soils in the period of 0–360 days

**Fig. 5:**
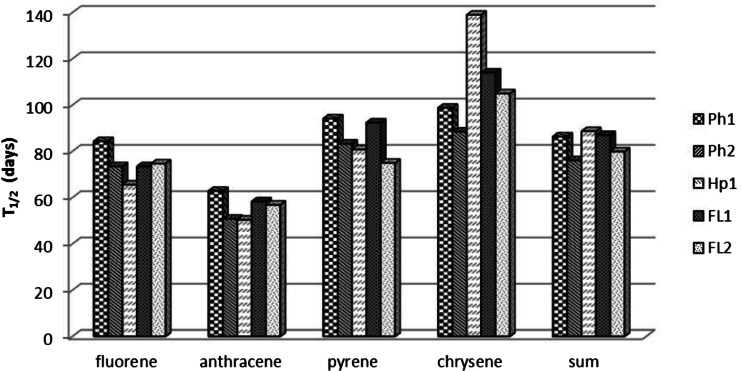
Half-life (*T*_1/2_) of the PAHs in soils in the period of 0–360 days

The lowest content of PAHs was recorded for the soil samples after 360 days of the experiment (Fig. 5). The content of anthracene ranged from 0.71 to 1.9 %, fluorene from 2.2 to 5.2 %, pyrene from 3.6 to 7.1 %, and chrysene from 6.0 to 16.5 %, as compared to the initial content (Table 4). The lowest rate of decomposition throughout the incubation period was found for chrysene. An average time of the half-life of this PAH in the examined soils was 109 days. The lowest *T*_1/2_ was received for anthracene, on average, 56 days. Therefore, the following order of decrease in stability of PAHs was observed in all of the soil samples:

$$ \mathrm{chrysene}>\mathrm{pyrene}>\mathrm{fluorene}>\mathrm{anthracene}. $$

The coefficients of correlation between the content of fluorene, anthracene, pyrene, and chrysene in the initial soil samples and the content of those PAHs in the soil samples after the completion of incubation show that after 360 days of decomposition, the content of anthracene, fluorene, and pyrene positively correlated with their initial content. No such relationship was found for chrysene, which, to some extent, can point to differences in the transformations of the hydrocarbons (Table 6).

**Table 6:** Significant values of the coefficients of correlation between the content of PAHs in the initial soil samples (0 days; “0”) and the PAH content in the soil samples after 360 days of incubation (“360”)

	Fluorene—“0”	Anthracene—“0”	Pyrene—“0”	Chrysene—“0”
Fluorene—“360”	0.7924			
Anthracene—“360”		0.9078		
Pyrene—“360”			0.7520	
Chrysene—“360”				−

**Fig. 6:**
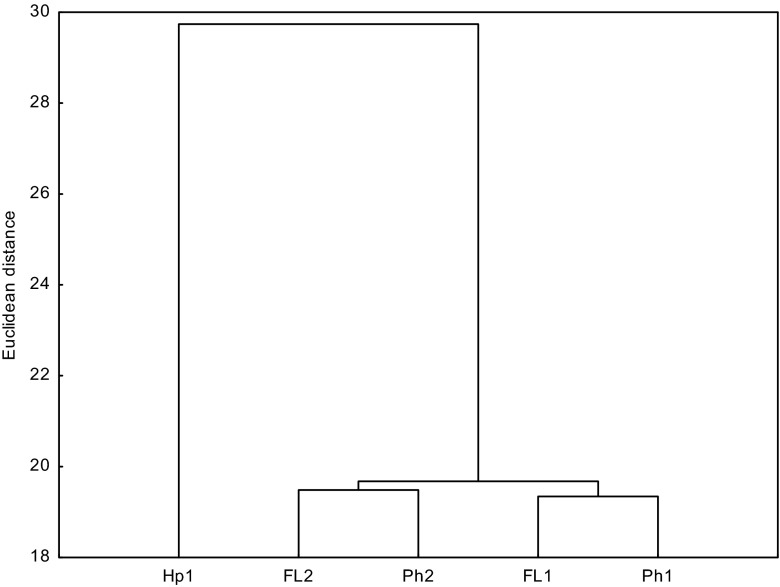
Cluster analysis of soils determined based on half-life (*T*_1/2_) of the PAHs calculated for the period of 0–360 days

The dendrogram pattern that was produced based on the half-life over 0–360 days indicates that those values are determined by the soil type (Fig. 6). The dendrogram shows one large group that is divided into two subgroups that included Gleyic Fluvisols with the texture of fine sand (FL2) and Gleyic Phaeozems with a lower content of TOC (Ph2) as well as *Mollic Fluvisols* with the texture of sandy loam (FL1) and Gleyic Phaeozems (Ph1). Haplic Arenosols were located outside those subgroups. One should stress that the effect of the soil type on the decomposition rate of PAHs was strongest over 30–180 days (Fig. 4), namely during the period in which most of PAHs that were introduced had already decomposed.

The relationships clearly point to the fact that in the process of the decomposition of PAHs that enter soil, the structure and the properties of PAHs themselves as well as the soil properties are essential. However, a single-time inflow of PAHs into soil at a high concentration results in a significant intensification of the decomposition rate of PAHs, which mostly depends on the properties of PAHs that are introduced into soil. After a longer period of the presence of PAHs in soil, the decomposition rate is mostly determined by soil properties, which can have an essential effect for soils that are continuously exposed to the effects of PAHs.

## Conclusions

1. The decomposition rate of the polycyclic aromatic hydrocarbons that were introduced into soils depended on the stage of the experiment. The degradation process of PAHs was most intensive during the first 30 days of incubation.
2. The intensity of the decomposition of PAHs in the initial incubation period depended on the properties of PAHs. A faster decomposition, as compared with pyrene and chrysene, was found for anthracene and fluorene, which are compounds with a lower molecular weight and a lower number of aromatic rings.
3. The correlations recorded between the basic soil properties and the half-life of PAHs show that the soil quality parameters are essential for the processes of the sorption and activation of PAHs.
